# The comparative effects of high fat diet or disturbed blood flow on glycocalyx integrity and vascular inflammation

**DOI:** 10.1186/s41231-018-0029-9

**Published:** 2018-11-22

**Authors:** Ronodeep Mitra, Ju Qiao, Sudharsan Madhavan, Gerard L. O’Neil, Bailey Ritchie, Praveen Kulkarni, Srinivas Sridhar, Anne L. van de Ven, Erica M. Cherry Kemmerling, Craig Ferris, James A. Hamilton, Eno E. Ebong

**Affiliations:** 1 Department of Bioengineering, College of Engineering, Northeastern University, Boston, MA, USA; 2 Department of Chemical Engineering, College of Engineering, Northeastern University, 360 Huntington Avenue, 313 Snell Engineering Building, Boston, MA 02115, USA; 3 Department of Mechanical and Industrial Engineering, College of Engineering, Northeastern University, Boston, MA, USA; 4 Department of Mechanical Engineering, College of Engineering, Tufts University, Medford, MA, USA; 5 Department of Biology, College of Science, Northeastern University, Boston, MA, USA; 6 Department of Psychology, College of Science, Northeastern University, Boston, MA, USA; 7 Department of Physics, College of Science, Northeastern University, Boston, MA, USA; 8 Department of Physiology and Biophysics, Boston University School of Medicine, Boston, MA, USA; 9 Department of Neuroscience, Albert Einstein College of Medicine, New York, NY, USA

**Keywords:** Glycocalyx, Endothelial dysfunction, Atherogenesis, Inflammation, High fat diet, Disturbed blood flow

## Abstract

**Background and aims:**

Endothelial surface glycocalyx shedding plays a role in endothelial dysfunction and increases vessel wall permeability, which can lead to inflammation and atherogenesis. We sought to elucidate whether a high fat diet (HFD) or disturbed blood flow conditions, both of which are atherogenic risk factors, would contribute more detrimentally to pre-atherosclerotic loss of endothelial glycocalyx integrity and vascular inflammation.

**Methods:**

Six to seven week-old C57BL/6-background apolipoprotein-E-knockout (ApoE-KO) male mice were either fed a chow diet, fed a modified Western HFD, and/or subjected to a partial left carotid artery (LCA) ligation procedure to induce disturbed blood flow patterns in the LCA. Mice were sacrificed after 1 week of experimental conditions. Both LCA and right carotid artery (RCA) vessels were dissected and preserved to compare glycocalyx coverage and thickness as well as macrophage accumulation in carotid arterial walls amongst and between cohorts.

**Results:**

Glycocalyx coverage of the endothelium was significantly reduced in the LCAs of HFD fed mice when compared to the control. More significant reduction in glycocalyx coverage occurred in the LCAs of mice exposed to disturbed flow by partial LCA ligation when compared to the control. No differences were found in glycocalyx coverage of RCAs from all cohorts. Regarding inflammation, no difference in macrophage accumulation in carotid arterial walls was observed when comparing the LCAs and RCAs of control and HFD fed mice. However, macrophage infiltration in vessel walls showed a 20-fold increase in the LCAs exposed to disturbed flow following ligation, when compared to control LCAs, while no such statistical difference was observed between the RCAs of the group.

**Conclusions:**

In our mouse model, endothelial glycocalyx integrity was compromised more by disturbed blood flow patterns than by exposure of the carotid vessel to HFD conditions. The pathophysiological implications include endothelial dysfunction, which correlates to macrophage infiltration in vessel walls and promotes atherogenesis.

## Background

The endothelial glycocalyx (GCX) acts as a buffer between the endothelium surface and blood flow-derived shear forces and contributes to vascular barrier functionality by limiting leakage of fluid and macromolecules across the endothelial cell layer. The endothelial GCX is a negatively charged, heavily hydrated, heterogeneous polysaccharide mesh layer located on the inner lumen, bound to the endothelial cell membrane [1, 2]. It largely consists of cell-linked sialic acid and glycosaminoglycans including heparan sulfate, hyaluronic acid, and chondroitin sulfate [3]. GCX integrity is dependent on blood chemistry and flow patterns along the walls of the vasculature.

The impact and influence of flow on the GCX have been demonstrated in previous studies. For example, it provides protection of the endothelium along vessel walls exposed to uniform blood and in relatively good health [2, 4]. However, in geometrically complex vasculature, vessel walls are exposed to non-uniform flow patterns with low wall shear stresses due to blood recirculation. In these vessels, endothelial cells exhibit rapid dysfunction, predisposing the vessels to endothelial-dependent atherogenesis.

Endothelial dysfunction and initiation of atherosclerosis propensity have been attributed to GCX thinning [5]. Van den Berg et al. [6] showed a thinned endothelial GCX at the bifurcation region of a mouse internal carotid artery compared to the common carotid artery [2] with impaired barrier properties of the GCX, contributing to enhanced pro-atherogenic low density lipoprotein (LDL) accumulation in the intima at the carotid artery bifurcation [7]. Their work has been corroborated and extended by others showing that local hemodynamics plays a vital role in GCX shedding and inflammation [8–10].

Fewer studies have shown the impact that a diet has on the integrity of the GCX. For instance, Dickinson et al. [11] showed that mice on a high salt diet exhibited increased responsiveness of arterial endothelial cells to pro-inflammatory cytokine (TNF*α*) at areas of disturbed blood flow within arteries. High salt diet also increased adhesion of leukocytes to these same areas of disturbed flow [12].

To date, no study has compared the impact of disturbed blood flow patterns versus a high fat diet (HFD) on GCX structure and ultimately its role in inflammation that precedes atherosclerotic plaque formation. We hypothesized that disturbed blood flow patterns would have a more profound and quicker impact on GCX integrity compared to a HFD. Thus, a major objective of this present work is to understand which risk factor, HFD or disturbed blood flow, has a more profound and quicker impact on GCX integrity and its functionality, as indicated by the extent to which endothelial cells permit inflammatory cell permeability in vessel walls. Although vessel wall inflammation precedes atherogenesis, studying the comparative effects of HFD versus disturbed blood flow on atherosclerotic plaque formation is outside of the scope of the present investigation.

To test our hypothesis, we performed in vivo experiments with apolipoprotein-E-knockout (ApoE-KO) mice exposed to a HFD ad libitum or exposed to acute disturbed blood flow in the left carotid artery (LCA) induced by performing a partial ligation surgery [13]. ApoE-KO mice have extensively been used as an animal model that develops extensive atherosclerotic lesions [14–16]. We quantified the GCX percent coverage and thickness of the inner vessel wall in the LCA and RCA for each mouse treatment condition. In addition, cluster of differentiation 68 (CD68) molecule was labeled by immunohistochemistry to mark macrophage presence within vessel walls. The results of this study suggest that blood flow patterns and not a HFD have a greater impact on vascular GCX integrity, hence correlating to more prominent dysfunctionality of the endothelium, a predecessor to atherogenesis.

## Methods

### Animal cohorts

All animals were conducted under protocol number 17-0824R, approved by the Northeastern University Institutional Animal Care and Use Committee (NU-IA-CUC). Five to six-week-old, male C57BL/6-background Apolipoprotein E-Knockout (ApoE-KO) mice were obtained from Jackson Laboratories. An acclimation period of 1 week was given to each mouse. During this one-week acclimation period, all mice were fed a regular chow diet and water ad libitum. Experimental conditions were initiated in mice between 6 and 7 weeks of age. Three cohorts of mice were tested, as summarized in Table 1: (I) ApoE-KO mice/chow diet/no LCA ligation, (II) ApoE-KO mice/HFD/no LCA ligation, and (III) ApoE-KO mice/chow diet/LCA ligation. Mice from cohort I with a normal diet and no surgery served as controls.

The HFD was a modified Western Diet with the addition of 3 g of added of cholesterol per kg (Research Diets, Inc. Product: D02031507) ad libitum for 1 week.

In previously published studies, sham surgery was performed, confirming that endothelial cell and vascular tissue responses do not occur due to incisional trauma alone [17, 18]. In the present surgery, an incision was accompanied by partial ligation of the LCA as previously described [13, 19], with minor modifications, to induce disturbed flow. Mice were placed on a Gaymar Heat Therapy Pump (Model #TP700) maintained at 38 °C, to stabilize the body temperature. Mice were anesthetized with 1.5–2.5% of isoflurane with a constant oxygen flow rate of 1 L/min. Puralube® ophthalmic ointment was used as a protective eye lubricant. Epilation of the ventral neck area of the mice was achieved using Nair™ and then disinfected using 70% ethanol followed by iodine. A surgical microscope was utilized to make a ventral midline incision (4–5 mm) in the neck and to locate and isolate the LCA by blunt dissection. Three of the four caudal branches of the LCA were ligated with 6–0 silk sutures. The superior thyroid artery was intact to assure minimal blood flow in the LCA. The incision was then closed with 5–0 nylon sutures and further reinforced with 3M Vetbond™ tissue adhesive. The RCA was not injured to provide a control in each mouse. Mice were monitored until recovery on a heated pad post-surgery. A single subcutaneous injection of Metacam (5 mg/mL) was administered immediately post-surgery for additional pain relief. Mice were monitored extensively for 3 days after surgery, to ensure proper recovery.

### Animal magnetic resonance imaging (MRI)

Magnetic resonance imaging (MRI) was performed at the Center of Translational Neuroimaging (CTNI) at Northeastern University in accordance with the Division of Laboratory Animal Medicine and Institutional Animal Care and Use Committee. MRI images were used to demonstrate blood volume in ligated LCAs (disturbed flow) as well as the counterpart RCAs that were not surgically ligated (uniform flow). MRI images were acquired at ambient temperature (25 °C) using a Bruker Biospec 7.0 T, 20 cm, scanner (Bruker, Billerica, Massachusetts, USA) equipped with a magnetic field gradient of 20 G per cm (ID = 12 cm, Bruker) and a 300 MHz, 3 cm diameter mouse coil (Animal Imaging Research, LLC, Holden, Massachusetts, USA). Ultra-short echo time (UTE) images were taken using a 3D-UTE pulse sequence with a 3 cm isotropic field of view (FOV) and 150 μm resolution, 200 × 200 × 200 μm matrix, echo time (TE) =13 μs, repetition time (TR) = 4 ms, flip angle (FA) = 20°, 200 kHz acquisition bandwidth, and a 200 kHz non-slice selective hard pulse for excitation [20]. After pre-contrast images were taken, mice intravenously received a bolus of 14 mg/kg of ferumoxytol (for approximately 200 μg/ml iron concentration in blood). Post-contrast UTE images were taken, rendered using 3D Slicer (Brigham and Women’s Hospital, Boston, MA) to show carotid arteries. All images were systematically obtained 1, 5, and 7 days post-partial ligation surgery.

### Characterization of LCA and RCA flow

From the UTE images, a 3D model of the subject-specific mouse aorta was constructed in the open-source computational fluid dynamics software, Simvascular [21]. First, centerline paths were created along the vessels. Next, 2D segmentation was performed at cross-sections spaced 5 mm apart along each of these paths. These segmentations were lofted to create solid models of the vessels. Boolean addition and blending were employed to combine the vessels and generate a smooth model. This procedure was performed on both pre-ligation (day 0) and post-ligation (day 1) images.

The model included the aortic arch, LCA, and RCA. The pre-ligation aorta, LCA, and RCA had mean diameters of 1.61 mm, 0.60 mm, and 0.57 mm respectively, and negligible vessel diameter change was assumed for the post-ligation model. A perspective view of the day ‘1’ model is shown in Additional file 1: Figure S1.

We employed the meshing tool that utilizes the Tetgen package [22], built-in to Simvascular [21], to discretize the subject-specific geometries using tetrahedral grid elements. The incompressible 3D Navier–Stokes equations were solved using the finite element formulation [23]. Pressure and velocity were coupled using an implicit scheme [24]. The non-linear residual of the Navier-Stokes equations was set to be less than 0.01 for convergence, meaning a maximum error of 0.01% should be expected in the solution due to convergence issues. Blood was modeled as a Newtonian fluid with a density of 1060 kg/m^3^ [25] and a viscosity of 0.0035 Pa·s, which is appropriate for the shear rates observed in mice [26]. Despite the fact that Newtonian models underestimate some physiological flow parameters, the qualitative aspects of Newtonian simulations of blood flow have been shown to be similar to those predicted by non-Newtonian models [27–31]. Previous studies have shown that the mean difference in wall shear stress between Newtonian and non-Newtonian models is about 10% [27, 30]. The vessel walls were assumed to be rigid, which we anticipate would contribute to less than 4.5% error in the solution [32].

In the current work, 3,022,013 tetrahedral elements were employed to discretize the geometry. A minimum element size of 0.0026 cm and a maximum element size of 0.095 cm were observed. Doubling the number of elements contributed to only about 2.7% root-mean-square (RMS) difference in terms of the velocity magnitude. A zoomed-in section of the grid is shown in Additional file 1: Figure S1B. Temporally, a second-order scheme with a time step of 0.01 s was employed, which was found to be both stable and efficient for our model in comparison to the other available options.

Additional file 2: Figure S2 illustrates a schematic of the model with boundary conditions. For the pre-ligation (day 0) model, a steady aortic inlet flowrate of 0.23 mL/s, based on the mean physiological aortic flow in mice aortas, was assumed [33, 34]. A parabolic inlet velocity profile was also assumed. Although the shape of the inlet velocity profile changes the local details of the flow near the aortic arch, it has been shown that such an assumption yields good accuracy for regions beyond two aortic inlet diameters downstream of the inlet [25, 35, 36]. Based on the bulk inlet velocity and the inlet diameter, the Reynolds number was 95. Resistance boundary conditions [37] were applied to the outlets as shown in Additional file 2: Figure S2. The resistance in the descending aorta was calculated by assuming a pressure drop from 98.2 mmHg at the ascending aorta inlet to 97.3 mmHg at the descending aorta outlet, as shown in Additional file 2: Figure S2A and 2D. These pressures were based on previously acquired pressure measurements in the mouse aorta [33]. The resistances for the common carotid outlets were determined from the pressure drops and flow rate splits [25]. The descending aorta, LCA, and RCA resistances are summarized in Table 2.

### LCA and RCA tissue preservation and cryostat sectioning

All mice were sacrificed via exsanguination 1 week after treatment. Mice were anesthetized with a single intraperitoneal injection of ketamine (100 mg/mL) and xylazine (20 mg/mL). Tissue fixation was performed by perfusing mice with phosphate buffer solution (PBS) containing 6.7% bovine serum albumin (BSA) and Ca^2+^ and Mg^2+^ adjusted to a pH of 7.2, followed by 2% paraformaldehyde (PFA) in PBS. Both LCA and RCA were then isolated and dissected for histology from the middle region (3–4 mm) between the aortic arch and below the bifurcation. This minimized the amount of edge effects associated with flow and shear stress patterns near the aortic arch inlets, bifurcation, and ligation sites. Arteries were embedded in Tissue-Tek optimum cutting temperature (OCT) medium and frozen in liquid nitrogen.

Arterial tissues, while embedded in OCT blocks, were transversely sectioned into rings of 6 *μ*m in thickness. Each tissue ring was transferred to a positively charged glass slide with three rings placed onto each slide. Sections were dried overnight and immediately stored at −80 °C with anhydrous calcium sulfate for desiccation, until further usage.

### Immunohistochemical staining

Platelet endothelial cell adhesion molecule (PECAM) was used to stain endothelial cells, while sialic acid and N-Acetyl-D glucosamine, a component of heparan sulfate and hyaluronic acid, were fluorescently tagged using wheat germ agglutinin (WGA) to stain for the GCX layer. For PECAM and WGA staining, LCA and RCA rings were post-fixed in 4% PFA in PBS for 10 min. PBS was used to thoroughly wash the fixative before the slides were permeabilized with 0.3% Triton diluted in PBS for an additional 10 min. Antigen retrieval was achieved by heating samples in pH of 6.0, 10 mM sodium citrate in PBS, for 10 min and placed on a bench top to equilibrate to room temperature. Endogenous peroxidase blocking was achieved by using a 1% hydrogen peroxide solution diluted in deionized water for 30 min. For PECAM staining, tissue samples were introduced to a 10% goat serum and a 0.3% triton blocking solution diluted in PBS for 1 h in a humidity-controlled chamber at 4 °C. Similarly, for WGA staining, tissue samples were introduced to a 5% BSA and a 0.3% triton blocking solution diluted in PBS for 1 h in a humidity-controlled chamber at 4 °C. Next, a 1:50 solution was created using either rabbit polyclonal anti-PECAM CD31 or biotinylated WGA, obtained from Vector Laboratories (Catalog No. B-1025) and diluted in its respective blocking solutions. Slides were incubated for 2 days in a humidity-controlled chamber at 4 °C. Following primary incubation, a 1:250 dilution of streptavidin-horseradish peroxidase (HRP) conjugate, from Thermo Fisher Scientific (Catalog No. SA10001), was diluted in PBS and incubated for 1 h in a controlled humidifier chamber at 4 °C. After, tissue samples were extensively washed in a 0.1% Tween 20 (Sigma-Aldrich Co.) in PBS solution. Next, a TSA Cyanine 3 amplification system kit from Perkin Elmer (Part No. NEL704A001KT) was used to incubate tissue samples for 5 min. Finally, the slides were again thoroughly washed in a 0.1% Tween 20 in PBS solution and mounted with Vectashield mounting medium from Vector Laboratories. The mounting medium contained 4′,6-diamidino-2-phenylindole (DAPI), a fluorescent stain that binds to adenine and thymine regions of DNA to mark the cell nuclei.

Similar steps were taken to identify macrophage infiltration in vessel walls using an immunofluorescent antibody against cluster of differentiation 68 (CD68). Tissue samples were post-fixed using a 4% PFA dilution in PBS for 10 min and then thoroughly washed in PBS immediately after. Slides were blocked in a 4% rabbit serum (Sigma-Aldrich Co.) dilution in PBS for 10 min. Then, 1:250 dilution of primary rat anti-mouse CD68 (Bio-Rad) in PBS containing 4% rabbit serum was incubated with the tissue sections in a humidity-controlled chamber at 4 °C for 1 h. Afterwards, the slides were thoroughly washed in a 0.1% Tween 20 (Sigma-Aldrich Co.) in PBS solution. Slides were then incubated for 10 min in a biotinylated rabbit anti-rat antibody, obtained from Vector Labs (Catalog No. BA-4001), diluted 1:200 in PBS containing 4% rabbit serum. The slides were again washed thoroughly in a 0.1% Tween 20 in PBS solution.

Following the washes, slides were incubated in a 1:500 dilution of HRP-conjugated streptavidin in 4% rabbit serum in PBS for 1 h in a humidity-controlled chamber. Finally, the slides were washed in 0.1% Tween 20 diluted in PBS. Tissue samples were treated for 5 min with the TSA Cyanine 3 amplification system kit from Perkin Elmer. Finally, samples were again thoroughly washed in a 0.1% Tween 20 in PBS solution. Slides were mounted with Vectashield mounting medium with DAPI and sealed with a cover sealant before imaging.

### Confocal microscopy imaging and data analysis

A Zeiss LSM 700 confocal microscope (Carl Zeiss Meditec AG, Jena, Germany), 20× and 63×-oil objectives, and excitation wavelength of 360 was utilized to visualize DAPI labeled cell nuclei of all vessel wall cells, a 488 wavelength was used to see elastin auto-fluorescence, and a 530 nm wavelength showed fluorescently labeled endothelial cells (PECAM), GCX (WGA), or macrophages (CD68).

GCX coverage and thickness and macrophage infiltration in the vessel wall were quantified using Fiji [38]. The fluorescence threshold was adjusted to eliminate or minimize background noise. To determine GCX coverage, a grid tool was used with the random offset function. Grid boxes that contained GCX covering the inner vessel wall were manually counted and compared with grid boxes that did not show GCX covering the entire inner vessel wall or in which GCX was completely absent. Equation (1) below was then used to approximate the percent coverage of the inner vessel wall by GCX:

(1) $$\%Coverage=\frac{\#boxes\;covering\;inner\;vessel\;wall-\#boxes\;with\;incomplete\;or\;absent\;GCX}{\#boxes\;covering\;inner\;vessel\;wall}\times 100$$

To limit the number of and randomize the grid boxes that needed to be analyzed for GCX thickness, each carotid artery tissue sample was divided into four different sections: top, bottom, right, and left area of the carotid artery (Additional file 3: Figure S3). Each grid box containing GCX was assigned a number using a MATLAB random number generator. Based on the random number generator, data points were obtained from three random grids in each sample section - top, bottom, right, and left. In total, 12 thickness data points were collected from each sample ring. Since there were three serial tissue rings on each slide, a total of 36 thickness measurements were obtained per LCA or RCA per animal. To do this, one transverse line was drawn on the GCX and measured (based on a calibration bar) in the middle of the designated grid box (Additional file 3: Figure S3).

To quantify the amount of macrophage infiltration, the vessel walls were imaged at 20× magnification. The fluorescence threshold was adjusted to eliminate or minimize background noise. The inner and outer vessel diameters were highlighted using a freehand tracing tool in FIJI (Fig. 4d). The percent area fraction of CD68 within the specified vessel wall boundaries was then determined.

### Statistics

Based on a post hoc power analysis conducted through G*Power version 3.1, we determined that a sample size of three mice would be adequate to test for statistical significance due to an achieved computed power of 0.9996, with an alpha value of 0.05. Values of GCX coverage, GCX thickness, and macrophage infiltration in LCA vessel walls were normalized to their RCA counterpart. The normalized results were expressed as mean ± standard error of the mean (SEM), and the results were plotted using GraphPad Prism. Normalized data sets were compared using a two-way ANOVA test to analyze statistical significance between groups with an alpha value of 0.05.

## Results

### Representative 3D MRI UTE images of disturbed flow conditioned mice

**Fig. 1:**
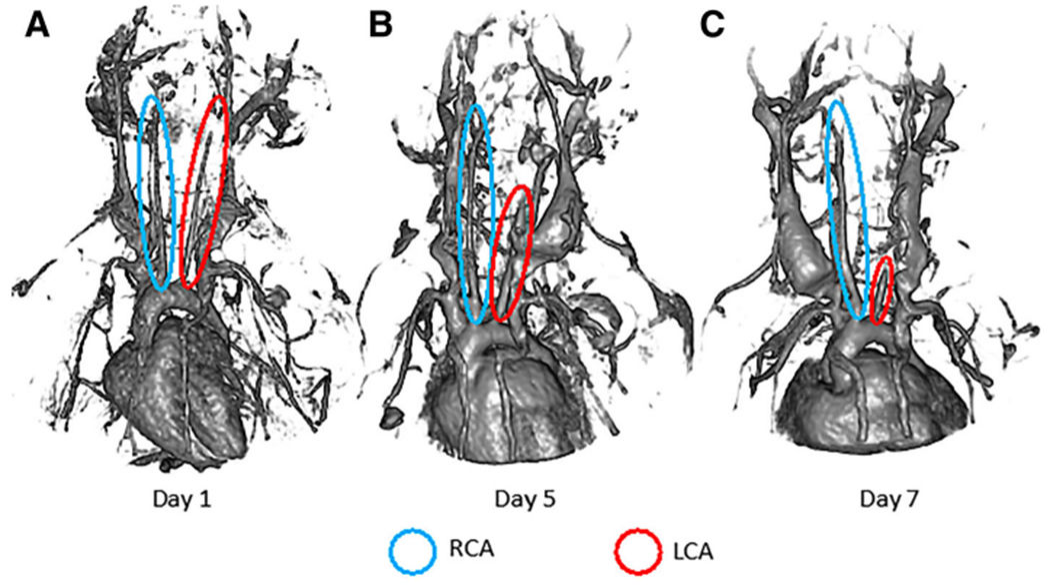
Representative UTE MRI images generated from detection of ferumoxytol flowing through vessels of mice in the disturbed flow conditioned experimental cohort, with blue circles indicating the RCAs and red circles indicates the LCAs. **a** Shows ferumoxytol-marked vascular structure of mice at day 1 post-ligation surgery. **b** The structure of visible vasculature is identified, revealed by ferumoxytol contrast, after 5 days post-ligation surgery. **c** The vascular integrity of the mouse after 7 days post-LCA ligation surgery is shown, as indicated by ferumoxytol contrast. Over time, reduction of detectable ferumoxytol in of the LCA suggests minimal blood flow, which would correspond to lower shear stress along the wall of the LCA. Additionally, there appears to be progressive remodeling of LCA vessel diameter and surrounding vessels, suggesting an inflammatory response, which is to be confirmed in the present study

MRI UTE images acquired from disturbed flow conditioned mice on days 1, 5, and 7 post-partial ligation procedure are presented in Fig. 1a-c. All images confirmed that ligation of the LCA induced disturbed blood flow. Iron-oxide nanoparticle ferumoxytol was used as the contrast agent, which acts as a blood pool agent at early time points. When comparing day 1 LCA (Fig. 1a) and day 7 LCA (Fig. 1c), the ferumoxytol in the LCA appears to span a shorter region of the LCA, indicating less blood flow in the LCA. Less blood flow suggests the presence of a reduced magnitude shear stress on LCA vessel walls, which was confirmed by analyzing the MRI UTE images using Simvascular flow simulations. Additionally, when comparing day 1 (Fig. 1a) and day 7 LCA (Fig. 1c) by inspection and qualitatively, there appears to be remodeling of LCA vessel diameter and surrounding vessels, suggesting an inflammatory response, which was confirmed by CD68 histology.

### Simulations of the experimental flow conditions in mice carotid arteries

**Fig. 2:**
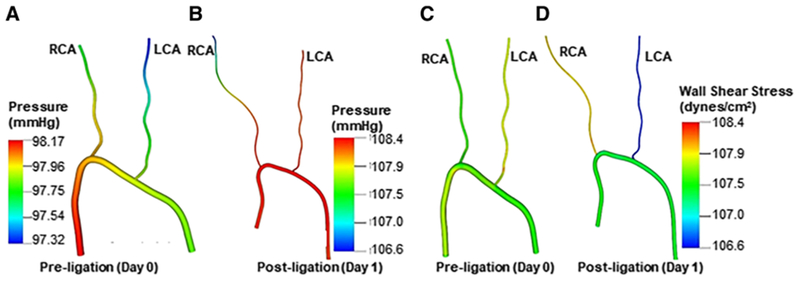
Pressure contour plots and wall shear stress from pre- and post-ligation mice. **a** Representative pressure contour plot of a pre-ligated mouse. **b** Representative pressure contour plot of a post-ligated mouse. **c** Representative wall shear stress contour plot from a pre-ligated mouse. **d** Representative wall shear stress contour plot from a post-ligated mouse

For all simulations, flow was assumed to be laminar since the Reynolds number based on the inlet diameter was 95. The flow simulations were performed for flow time equivalent to five murine cardiac cycles. Flow patterns, wall shear stresses, and pressures were extracted for pre- and post-ligation conditions. Table 3 summarizes the normalized wall shear stresses in the LCA and RCA. Both shear stresses and pressures are presented in Fig. 2.

To obtain these results, three vessel diameters were extracted from the middle section of the LCA and RCA. A wall shear stress as defined in Eq. (2) was computed for the pre-ligation (day 0) model by averaging the wall shear stress and acquiring the mean wall shear stress ($\bar{\mathrm{WSS}}$) in sections from both the RCA and LCA, resulting in 236.3 dynes/cm^2^.

(2) $$\bar{WSS}=\frac{\int_{wall}\mathrm{WSS}\cdot dA}{\int_{wall}dA}$$

The data was normalized with the $\bar{\mathrm{WSS}}$ as described in Eq. (3).

(3) $$\mathrm{Normalized}\;\mathrm{WSS}=\frac{\mathrm{WSS}}{\bar{WSS}}$$

Post-ligation, normalized wall shear stress increases in the RCA and decreases in the LCA. This is due to the highly-restricted post-ligation flow through the LCA. A similar trend is present in flow rate, as shown by Nam et al. [13].

Fig. 2 illustrates the pressure drop in the pre- (Fig. 2a) and post-ligation (Fig. 2b) models. A maximum pressure drop of 1.81 mmHg was calculated in the post-ligation model, a 113% increase in comparison to the 0.85 mmHg pressure drop calculated in the pre-ligation model. Additionally, the LCA pressure increased by approximately 11% when comparing pre- and post-ligation. The increase in LCA pressure drop from day 0 to day 1 can be attributed to the large increase in downstream resistance of the LCA, which severely restricts the flow entering this branch. The pressure in the ascending aorta also increased by about 10.5% in the post-ligation data, which was expected due to the restriction of the LCA, causing the heart to pump harder to force flow through the vessels with increased total vascular resistance.

Fig. 2c and d show the wall shear stress in pre- and post-ligation models. Overall, the LCA exhibited slightly higher overall wall shear stress in comparison to the RCA in the pre-ligation model (Fig. 2c). However, wall shear stress was significantly reduced in the LCA (Fig. 2d) post-ligation model, which is expected due to the decreased flow rate in the LCA.

Hence, flow simulations adequately characterize flow patterns, pressure, and wall shear stress when comparing the pre- and post-ligation mice. This confirms the notion that the LCA partial ligation surgery does indeed reduce wall shear stress and increase pressure, all characteristics of disturbed flow [29].

### WGA staining shows disturbed flow induces the most significant drop in GCX coverage on intact endothelium in the carotid arteries

**Fig. 3:**
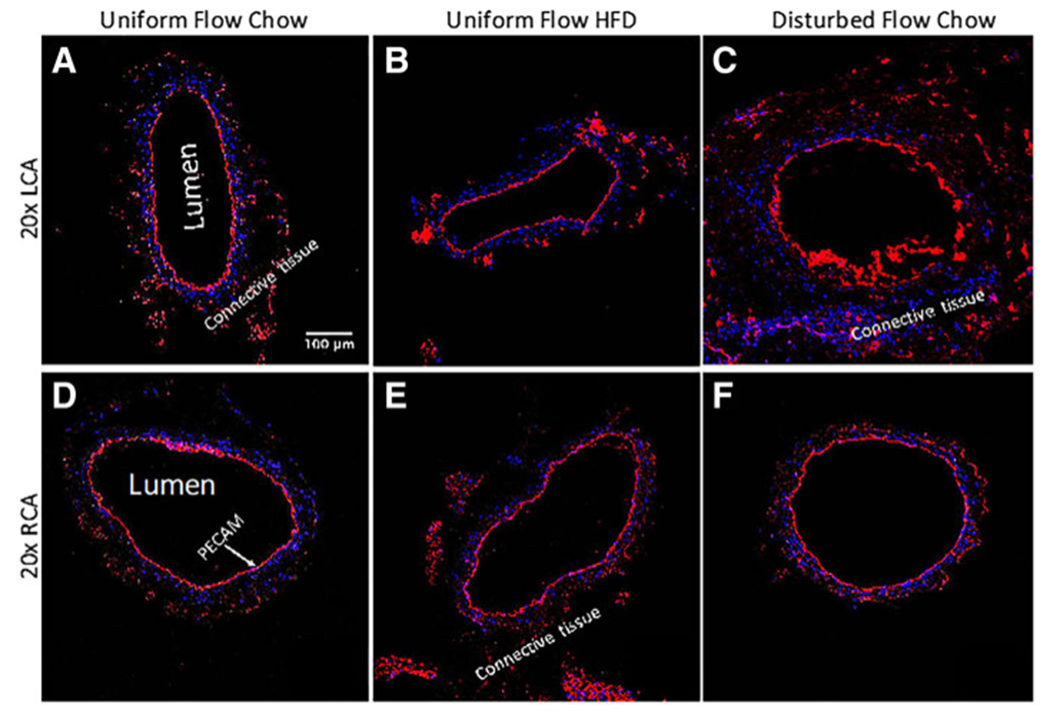
LCA and RCA vessels are immunostained for PECAM (red) to demonstrate the integrity of the endothelial cell layers on the inner vessel walls. Blue signifies DAPI labeled cell nuclei. **a** 20× image of the LCA obtained from an ApoE-KO mouse with a regular chow diet and exposed to uniform blood flow patterns. **b** 20× image of the LCA from an ApoE-KO mouse fed an HFD and exposed to uniform flow. **c** 20× image of the LCA from an ApoE-KO mouse on a regular chow diet and exposed to disturbed blood flow due to partial ligation. **d** 20× image of the RCA obtained from an ApoE-KO mouse with a regular chow diet. This RCA was exposed to uniform blood flow while its corresponding LCA was also exposed to uniform blood flow. **e** 20× image of the RCA from an ApoE-KO mouse on an HFD. This RCA was exposed to uniform blood flow while its corresponding LCA was also exposed to uniform blood flow. Finally, **f** 20× image of the RCA from an ApoE-KO mouse on a regular chow diet. This RCA was exposed to uniform blood flow while its corresponding LCA was exposed to disturbed blood flow

**Fig. 4:**
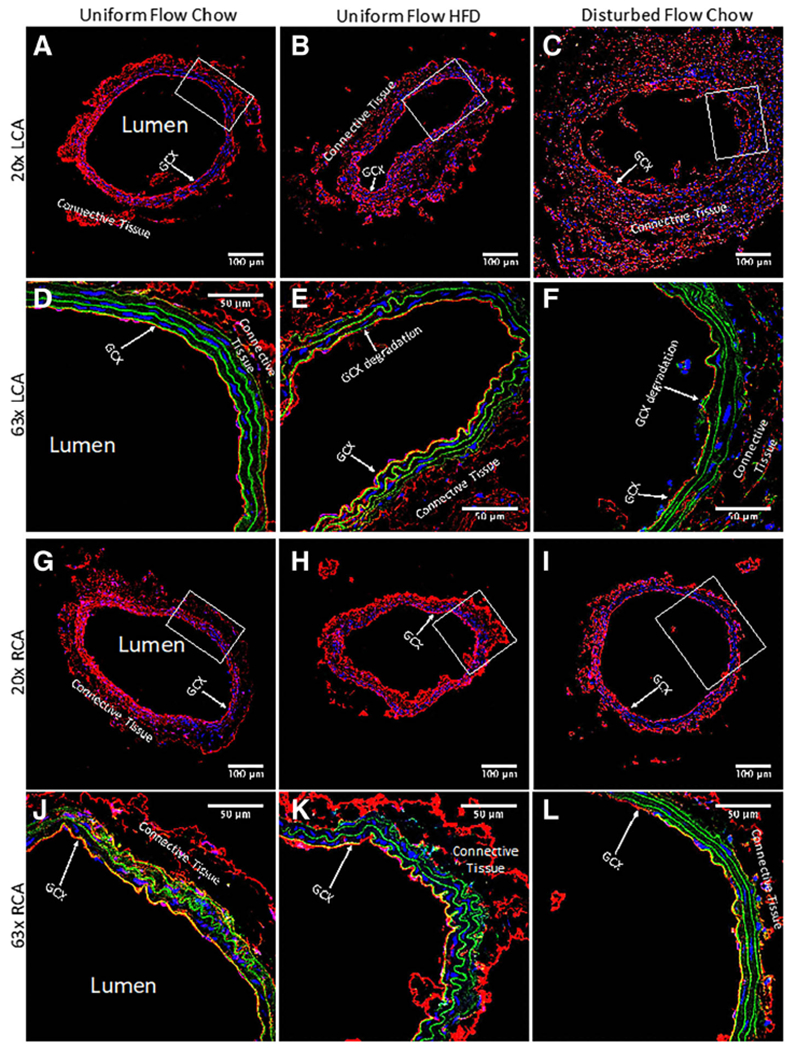
GCX in carotid artery rings of different mice cohorts was stained with WGA tagged with red fluorescence. Blue signifies DAPI labeled cell nuclei and green signifies elastin autofluorescence. These confocal images are representatives of arterial rings in the cohorts. **a** 20× image of the LCA obtained from an ApoE-KO mouse with a regular chow diet and exposed to uniform blood flow patterns. **b** 20× image of the LCA from an ApoE-KO mouse fed a HFD and exposed to uniform flow. **c** 20× image of the LCA from an ApoE-KO mouse on a regular chow diet and exposed to disturbed blood flow due to partial ligation. **d** 63× image of (**a**) zoomed in on the boxed area, showing a continuous GCX layer. **e** 63× image of (**b**) zoomed in on the boxed area, showing a discontinuous GCX layer. **f** 63× image of (**c**) zoomed in on the boxed area also, also showing a compromised GCX layer. **g** 20× image of the RCA obtained from an ApoE-KO mouse with a regular chow diet. This RCA was exposed to uniform blood flow while its corresponding LCA was also exposed to uniform blood flow. **h** 20× image of the RCA from an ApoE-KO mouse on a HFD. This RCA was exposed to uniform blood flow while its corresponding LCA was also exposed to uniform blood flow. Finally, **i** 20× image of the RCA from an ApoE-KO mouse on a regular chow diet. This RCA was exposed to uniform blood flow while its corresponding LCA was exposed to disturbed blood flow. **j** 63× image of (**g**) zoomed in on the boxed area, demonstrating a continuous GCX layer. **k** 63× image of (**h**) zoomed in on the boxed area, showing a continuous GCX layer. **l** 63× image of (**i**) in the designated boxed area also, showing a continuous GCX layer

As previously mentioned, sham studies conducted by Korshunov et al. [18] demonstrated no morphological differences between sham surgery carotid arteries and LCAs that underwent ligation surgery. Briefly, Korshunov et al. [18] observed no neointima formation in sham mice with non-ligated LCA, indicating that the suture material itself did not cause vessel remodeling [18]. Additionally, Go et al. [17] also implemented the same partial ligation surgery on the LCA and had a sham group. They demonstrated that the endothelium on the lumen side of non-ligated LCA in sham mice was intact, indicating that the surgical trauma does not damage the endothelium. In the present study, carotid arteries were stained to label PECAM, which confirmed that the endothelial layer remained intact for all experimental conditions (Fig. 3), with morphological appearance of endothelial dysfunction occurring in ligated LCA of ApoE-KO mice on a chow diet (Fig. 3c). This apparent dysfunction corresponded to observed changes in GCX, which were revealed by 20× and 63×-oil magnified visualization of WGA staining in LCA and RCA samples from each cohort of mice (Fig. 4).

As shown in the representative images of Fig. 4, GCX appeared fully intact and GCX percent coverage was significantly higher in the non-ligated LCA of ApoE-KO mice on a chow diet, in comparison to both non-ligated LCA of ApoE-KO mice on HFD and ligated LCA of ApoE-KO mice on a chow diet (Fig. 4a – f and Fig. 6a – b). GCX percent coverage was 84.8 ± 1.45% in the non-ligated LCA of chow diet fed mice (Fig. 6a – b). GCX percent coverage decreased by approximately 20% to 68.2 ± 2.40% in the non-ligated LCA of HFD fed mice (Fig. 6a – b), indicating diet-induced GCX degradation. Additionally, an approximate 50% decrease in GCX percent coverage to 41.2 ± 4.41% in the ligated LCA of chow diet fed mice was observed in comparison to the non-ligated LCA of chow diet fed mice (Fig. 6a – b), indicating substantial disturbed flow induced GCX degradation. The GCX percent coverage decreased by 27% when comparing the LCAs of non-ligated HFD mice to ligated chow diet mice (Fig. 6a – b).

GCX percent coverage was 65.9 ± 6.37% in the RCA of chow diet fed mice with a non-ligated LCA (Fig. 4g and j and Fig. 6a – b). This percent coverage was surprising, as it was statistically significant 22% lower than the coverage for the LCA GCX in the same cohort of mice. Additionally, GCX percent coverage was 61.7 ± 3.27% in the RCA of HFD fed mice with a non-ligated LCA (Fig. 4h and k and Fig. 6a – b), a smaller decrease in GCX percent coverage compared to the LCA GCX in the same cohort of mice. The GCX percent coverage was 50.8 ± 3.41% in the RCA of chow diet fed mice with a ligated LCA (Fig. 4i and l and Fig. 6a – b), a slight increase in GCX percent coverage compared to its LCA GCX in the same cohort of mice. In summary, there was no statistical difference found when comparing RCA GCX percent coverage of all cohorts (Fig. 6a – b).

The RCA data was used to normalize the LCA data. When normalizing the LCA results by respective RCA results, the normalized GCX coverage was 1.32 ± 0.155 in the non-ligated LCA of chow diet fed mice (Fig. 6e). Normalized LCA GCX coverage decreased by 15% in non-ligated LCA of HFD fed (Fig. 6e). Additionally, in the ligated LCA of chow diet fed mice, normalized GCX coverage showed the greatest decrease of approximately 40% (Fig. 6e).

### WGA staining shows no significant change in GCX thickness due to diet or due to flow conditions

GCX thickness for the LCA in all cohorts ranged between 2.45 and 2.89 *μ*m (Fig. 4a – f and Fig. 6a, c). Specifically, the GCX thickness in non-ligated LCA of ApoE-KO mice on a chow diet was 2.81 ± 0.029 *μ*m (Fig. 6a, c). Additionally, the GCX thickness in non-ligated LCA of ApoE-KO mice on a HFD was 2.89± 0.194 *μ*m (Fig. 6a, c). Finally, a slight decrease in GCX thickness to 2.45 ± 0.197 *μ*m was observed in ligated LCA of ApoE-KO mice on a chow diet (Fig. 6a, c).

GCX thickness for the RCA in all cohorts ranged from 2.60 to 2.89 *μ*m. The GCX thickness in the RCA of ApoE-KO mice with a non-ligated LCA on a chow diet was 2.89 ± 0.085 *μ*m (Fig. 4g and j and Fig. 6a, c). The GCX thickness in the RCA of ApoE-KO mice with a non-ligated LCA on a HFD was 3.16 ± 0.216 *μ*m (Fig. 4h and k and Fig. 6a, c), a slight increase from its LCA counterpart. Finally, the GCX thickness in the RCA of ApoE-KO mice with a ligated LCA on a chow diet was 2.60 ± 0.103 *μ*m, a slight increase from its LCA counterpart (Fig. 4i and l and Fig. 6a, c). However, no statistical difference was found when comparing RCA GCX thickness for all cohorts (Fig. 6a, c).

Normalization of LCA GCX thickness data by the RCA GCX thickness data presented a thickness range of 0.923 to 0.973, which indicates that the LCA and RCA GCX thicknesses are very comparable (Fig. 6f). No statistical differences were seen when comparing thicknesses of GCX in LCAs and RCAs from different cohorts of mice (Fig. 6f).

### CD68 staining of the carotid arteries, to assess inflammation, reveals that high inflammation corresponds to conditions of flow induced GCX degradation

**Fig. 5:**
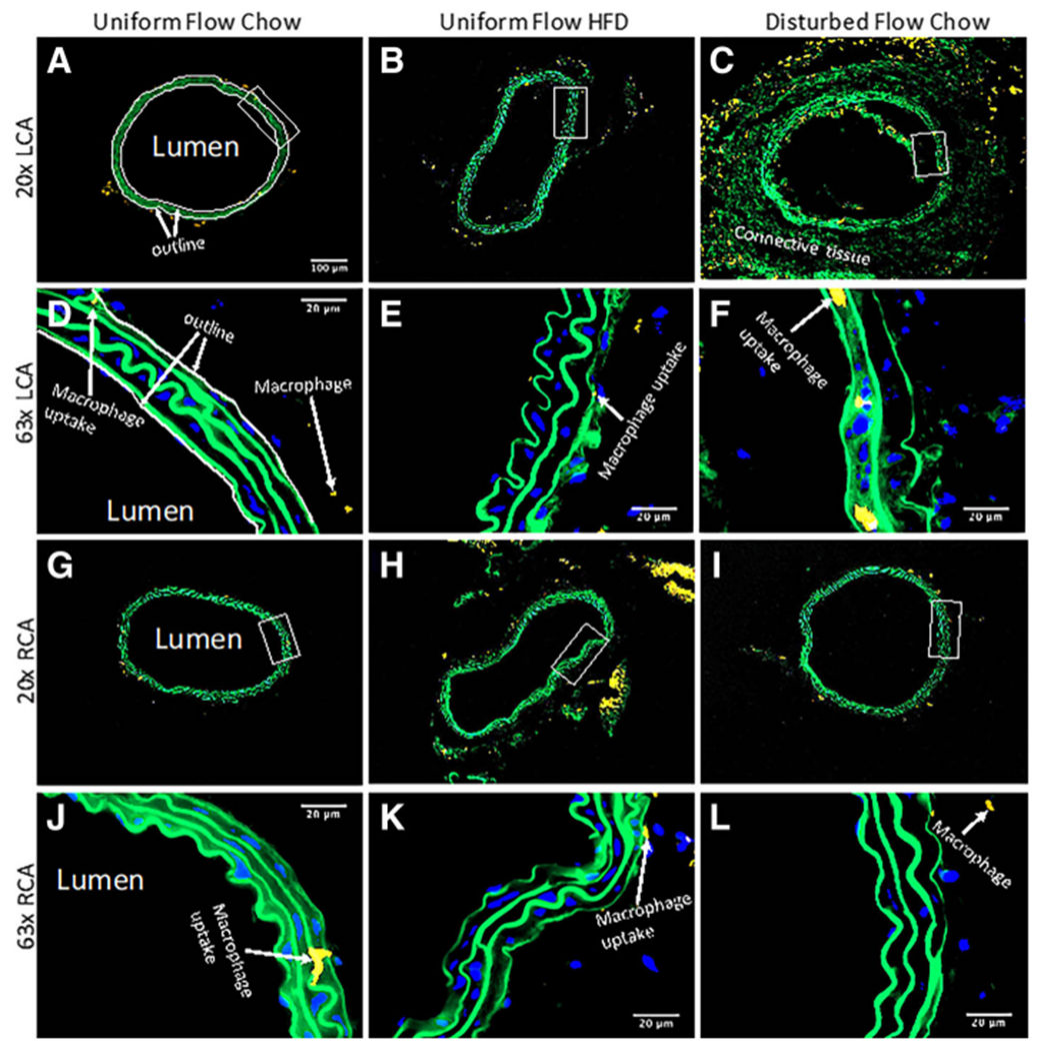
Visualization of yellow fluorescence tagged CD68-positive inflammatory cells, which infiltrated into carotid artery walls (see green autofluorescence of the elastin wall) to a greater extent in induced disturbed blood flow conditions in comparison to HFD conditions. Blue signifies DAPI, which labels the cell nuclei. From full sets of images like these, percent area fraction of macrophage deposition was calculated in carotid artery walls, which was then normalized by the RCA to its LCA counterpart. Carotid artery walls were marked by outlining the inner and outer diameter of the arterial walls, as demonstrated in (**a** and **d**). **a** 20× image of the LCA obtained from an ApoE-KO mouse with a regular chow diet and exposed to uniform blood flow patterns. **b** 20× image of the LCA from an ApoE-KO mouse fed an HFD and exposed to uniform flow. **c** 20× image of the LCA from an ApoE-KO mouse on a regular chow diet and exposed to disturbed blood flow due to partial ligation. **d** 63× image of (**a**) zoomed in on the boxed area, showing a continuous minimal macrophage deposition. **e** 63× image of (**b**) zoomed in on the boxed area, showing minimal macrophage deposition. **f** 63× image of (**c**) zoomed in on the boxed area also, showing an increased amount of macrophage deposition in arterial walls. **g** 20× image of the RCA obtained from an ApoE-KO mouse with a regular chow diet. This RCA was exposed to uniform blood flow while its corresponding LCA was also exposed to uniform blood flow. **h** 20× image of the RCA from an ApoE-KO mouse on an HFD. This RCA was exposed to uniform blood flow while its corresponding LCA was also exposed to uniform blood flow. Finally, **i** 20× image of the RCA from an ApoE-KO mouse on a regular chow diet. This RCA was exposed to uniform blood flow while its corresponding LCA was exposed to disturbed blood flow. **j** 63× image of (**g**) zoomed in on the boxed area, showing minimal macrophage deposition. **k** 63× image of (**h**) zoomed in on the boxed area, showing minimal macrophage deposition. **l** 63× image of (**i**) in the designated boxed area also, showing minimal macrophage infiltration

**Fig. 6:**
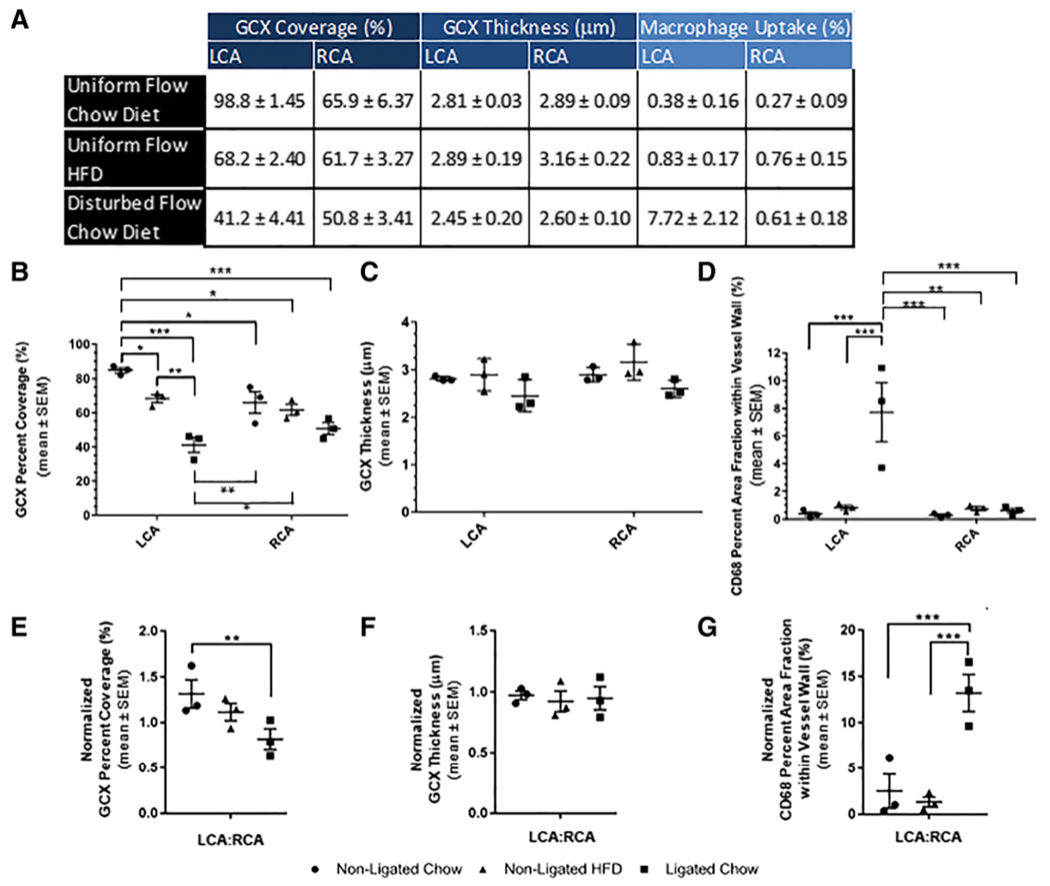
**a** Table shows the mean ± SEM GCX (Fig. 4) and macrophage infiltration (Fig. 5) data that was calculated from each cohort (which each include *N* = 3). **b** GCX coverage of inner vessel walls (LCA and RCA). **c** GCX thickness on inner vesselwalls (LCA and RCA). **d** Macrophage infiltration within LCA and RCA vessel walls. **e** Normalized GCX coverage of inner LCA walls (LCA:RCA). **f** Normalized GCX thickness on inner LCA walls (LCA:RCA). **g** Normalized macrophage infiltration within LCA vessel walls (LCA:RCA). **b**-**g** Each data point on graphs represents the mean for individual ApoE-KO mice in each group, where each group had *N* = 3 mice. For each condition, the mean along with error bars indicating SEM, for all animals taken together, is also shown. Statistical significance was assessed by a 2-way ANOVA and is indicated by asterisks as follows: *p < 0.05, **p < 0.01, ***p < 0.001, and ****p < 0.0001

Figure 5 shows representative images of CD68 immunofluorescence, which identifies macrophages embedded in the walls of carotid arteries amongst all cohorts. A percent area fraction was calculated to determine macrophage infiltration within vessel walls of carotid arteries. Macrophage infiltration in the ligated LCA of chow diet fed mice was determined to be 7.72 ± 2.12%, a substantial 20-fold increase when compared to the non-ligated LCA of chow diet fed mice, with infiltration of macrophages into 0.375 ± 0.163% of the vessel wall (Fig. 5a and c, Fig. 6a, d). A 10-fold increase was also observed when comparing macrophage infiltration in ligated LCA of chow diet fed mice to macrophage infiltration in the non-ligated LCA of HFD fed mice, which was only 0.828 ± 0.166% (Fig. 5b and c, Fig. 6a, d). Normalized macrophage infiltration data showed similar trends, with a 5-fold increase for conditions of ligated LCA in chow diet fed mice compared to non-ligated LCA in chow diet fed mice (Fig. 6a, g).

Macrophage infiltration in the RCA corresponding to the animals with non-ligated LCA and chow diet was determined to be 0.271 ± 0.085%, which was comparable to the macrophage infiltration in its LCA counterpart (Fig. 5a and g, Fig. 6a, d). Additionally, macrophage infiltration in the RCA of mice with non-ligated LCA and fed a HFD was 0.755 ± 0.154%, which was also comparable to the macrophage infiltration in its LCA counterpart (Fig. 5b and h, Fig. 6a, d). The macrophage infiltration in the RCA from mice with ligated LCA and fed a chow diet was 0.607 ± 0.178% (Fig. 5i, Fig. 6a, d). When comparing the normalized macrophage infiltration results for the ligated LCA in the chow diet fed mice to the RCA from the same mice, there is a statistically significant 13-fold increase in macrophage vessel wall infiltration (Fig. 5c and i, Fig. 6g). No other such statistical significance was encountered when comparing the LCAs and RCAs within each of the other two cohorts, control and HFD fed mice.

### Summary of pathological findings

The results acquired from the experimental cohorts, including endothelial GCX coverage and thickness, and macrophage infiltration within vessel walls, are summarized in Fig. 6.

## Discussion

In the present study, 1 week after partial LCA ligation, ApoE-KO mice exhibited intact endothelium but scarce GCX on the inner LCA wall. This GCX scarcity is a direct consequence of multidirectional, low magnitude, and oscillatory shear stress caused by the ligation procedure. Typically, uniform flow conditions [5] and intact GCX are present in the common LCA. Previously, GCX has only been found to be degraded in vascular regions of naturally occurring disturbed flow, such as the carotid artery bifurcation [7] and the brachiocephalic artery region that lies adjacent to its right subclavian artery and right common carotid artery branches [5]. However, in the present study, the GCX damage is more extensive, consistent with the widespread flow disturbance induced by the partial LCA ligation as demonstrated through MRI and Simvascular flow simulations. In addition, low GCX expression was observed in non-ligated LCA of ApoE-KO mice when the mice were fed a HFD for 1 week. However, this HFD-induced reduction in GCX expression was not as severe as what we found in response to partial LCA ligation after 1 week. In Cancel et al. [5], we previously studied the effects of simultaneous HFD and natural disturbed blood flow conditions on GCX integrity and endothelial dysfunction. We found HFD fed mice to exhibit significantly reduced GCX, which coincided with endothelial dysfunction, on atherosclerotic plaques in disturbed flow conditioned regions of the brachiocephalic artery [5]. GCX was abundant on non-plaque vessel walls in uniform flow conditioned regions of the brachiocephalic artery of the same HFD fed mice [5]. The previous study [5] led us to wonder if GCX degradation occurs as a consequence of the atherosclerosis process or if GCX degradation precedes atherosclerosis. Furthermore, if GCX degradation precedes atherosclerosis, we questioned whether the degradation was due to HFD or due to disturbed flow conditions. To our knowledge, no study has examined the individual effects of HFD versus disturbed blood flow on GCX degradation. The present study is the first clear demonstration that non-uniform blood flow, when compared to fatty diet, has a more detrimental impact on the integrity of the endothelial GCX.

The large decrease in GCX coverage of inner vessel walls has significant pathophysiological implications. LDL leakage across diseased vessel walls and increased macrophage infiltration in vessel walls are hallmarks of atherogenic progression. The anti-adhesive property of the GCX protects endothelial cells from inflammatory activation and impedes lipids and leukocytes from directly interacting with endothelial cells. Shedding of the GCX is thought to have a two-fold effect. One effect is that it allows for closer interaction between LDL and leukocytes, which activates endothelial cells and enhances the expression of inflammatory biomarkers. Another effect is that the absence of GCX exposes adhesive receptors that lie close to the endothelium surface, like low density lipoprotein receptor (LDL-R), vascular cell adhesion molecule 1 (VCAM-1), intercellular cell adhesion molecule-1 (ICAM-1), E-selectin, and P-selectin, enhancing inflammatory adhesion [1].

In support of our interpretations above, as shown in a short-term study conducted by van den Berg et al. [7], for the carotid artery bifurcation, GCX degradation can lead to impairment of endothelial barrier function and trans-endothelial leakage of atherogenic LDL. Our colleagues and us [5] showed in a longer (10-week) study in mice that brachiocephalic artery GCX degradation correlated to substantial lipid infiltration leading to atherosclerotic plaque formation. Li et al. [39] showed that damage to the GCX favors pathological adhesion and infiltration of macrophages into the vascular wall. Nagy et al. [40] demonstrated that inhibiting synthesis of hyaluronan, a major component of the GCX, using 4-methylumbelliferone reduced endothelial GCX and caused increased leukocyte-to-vessel wall adhesion and macrophage retention in the vessel wall. Similarly, Voyvodic et al. [41] showed increased leukocyte recruitment and inflammatory response in mice deficient in syndecan-1, a core protein of the GCX. In the present study, we found results that were consistent with those that were previously reported. In just 1 week after partial ligation of the LCA in chow diet fed ApoE-KO mice, a high degree of macrophage infiltration into vessel walls was observed in correlation to and likely as a result of the extremely limited extent of GCX expression. We speculate that beyond 1 week of exposure to flow disturbance, due to further anticipated decrease in GCX coverage of inner vessel walls, grave break down in the barrier against further infiltration of inflammatory cells will occur, causing additional damage and accelerated growth of atherosclerotic lesion [39].

We report two unexpected results from our study. First, the percent GCX coverage of RCA inner walls was relatively lower when compared to their counterpart LCA inner walls, in all cohorts. In control conditions, for the cohort of mice that were exposed to chow diet and uniform flow conditions (no partial ligation) in the LCA, the GCX covered 85% of the LCA walls while the GCX covered 66% of the RCA walls. Reitsma et al. [42] studied GCX coverage with WGA staining in the common carotid artery and established that GCX covered 80% of the LCA, giving us confidence in our LCA results. We think that the observed differences between GCX coverage of LCA versus RCA walls can be explained by the work of Sui et al. [43], who analyzed flow patterns and wall shear stresses in internal carotid arteries of humans using phase-contrast magnetic resonance sequence combined with a three-dimensional parabolic model. Sui et al. [43] concluded that flow patterns are variant and complicated at the origin of carotid arteries, and different types of local wall shear stress spatial distributions exist around the vessel circumference [43]. In fact, their data suggested that RCA vessel circumference area encounters low and oscillatory wall shear stress more often during systole and diastole compared to the LCA. Similar findings were established in our flow simulations, suggesting that the wall shear stress is generally low in the RCA compared to the LCA (Fig. 2c). Well-known risk factors for atherosclerosis occur preferentially at particular areas of disturbed flow characterized by low and oscillatory wall shear stress [44, 45]. Therefore, the fact that blood flow rates and wall shear stress patterns in the LCA and RCA are not equivocal may explain our observation that the GCX is diminished more in the RCA than LCA.

Another explanation for the lower percent of RCA inner wall coverage by GCX, as compared to their counterpart LCA inner walls, could be the fact that WGA only labels sialic acid and N-Acetyl-D glucosamine, a component of heparan sulfate and hyaluronic acid. WGA does not completely identify heparan sulfate and hyaluronic acid, and it does not stain chondroitin sulfate at all. Due to this, it is entirely possible that the RCA GCX may have more expression of other GCX components that our study did not detect. In fact, in chow diet fed mice that underwent the partial LCA ligation surgery, we observed an insignificant difference between the GCX coverage of both the LCA and RCA vessels, but macrophages did not infiltrate the RCA vessels. This confirms that the RCA does express more vasculoprotective GCX than we detected.

A second unexpected result was the preservation of consistent GCX thickness across all cohorts, although we expected that diminished GCX thickness would occur in the LCA under disturbed flow conditions. Our reported GCX thickness from all cohorts ranged from 2.45 to 3.16 *μ*m and no statistical significance was encountered when comparing the GCX thickness between cohorts. GCX thicknesses were comparable to previous studies [7, 46]. However, our colleagues and we [5] previously saw a significant decrease in GCX thickness when comparing non-plaque and plaque regions of the brachiocephalic artery. In our present study, no plaque was present in the carotid arteries due to the short lifespan of each mouse. Reitsma et al. [46] witnessed an absence of growth of the GCX in hyperlipidemic mice while GCX thickness increased in wild-type mice with age. The overall GCX thickness average was found to be 2.8 *μ*m. This endothelial GCX thickness still largely exceeded the size of adhesion molecules expressed on the endothelium like P-selectin [47, 48]. The question arises as to how macrophages interact with the endothelium despite such a protruding barrier. The explanation is that endothelial GCX thickness described in this study represents a central measurement derived from a vessel with GCX of heterogeneous thickness. Hence, especially in cases where GCX coverage of the endothelium is reduced, leukocytes do not have to overcome the entire GCX thickness segment but adhere and penetrate to vessel walls at endothelium sites that are covered with thinner GCX or not covered at all.

In ongoing studies, we are performing analysis of time points prior to and beyond 1 week using serial magnetic resonance imaging of the carotid arteries. We intend to also perform the partial ligation procedure on the RCA as an alternative, and in comparison, to the partial LCA ligation, accounting for the differences in blood flow patterns and wall shear stresses in the RCA versus the LCA. These ongoing and future studies will provide insight regarding spatial and temporal GCX thinning, macrophage infiltration, whole vessel wall remodeling, and atherosclerotic plaque formation in real time. Other inflammatory biomarkers can also be probed for further clarification of the importance of flow versus diet factors in atherogenesis. These additional studies will be the subject of future reports.

## Conclusion

We have gained valuable insight from our current measurements of GCX coverage and macrophage infiltration in vessel walls of the ApoE-KO mice on a chow or a 1-week HFD and with uniform or 1-week disturbed flow LCA conditions. In summary, our current studies showed that disturbed flow-induced damage to the GCX is more prominent than HFD-induced GCX damage, correlating to significantly higher macrophage infiltration in ligated LCA conditions in comparison to HFD conditions. These findings show that disturbed flow is a critical factor in correlating GCX degeneration upstream of endothelial cell dysfunction and eventual atherogenesis.

## Supplementary Material

Captions for Figures S1, S2, and S3

Figure S1 **Additional file 1: Figure S1.** (A) Perspective view of the discretized, image-based model of the subject-specific vasculature from 1-day LCA post-ligation surgery. (B) Zoomed in view of the computational mesh created. (JPG 94 kb)

Figure S2 **Additional file 2: Figure S2.** A schematic of the model from a preligation mouse with corresponding boundary conditions. (A) Parabolic inlet velocity profile. (B), (C), and (D) are resistance outlet conditions at RCA, LCA, and descending aorta respectively. (JPG 165 kb)

Figure S3 **Additional file 3: Figure S3.** 20× magnification images of the carotid arteries were used to determine GCX coverage and thickness. The red indicates GCX and the blue represent cell nuclei (DAPI). (A) The initial step was to count the total number of grid boxes encompassing the inner diameter (lumen side) of the arterial wall. Next, the number of grid boxes that contained an incomplete or absent GCX layer was tallied. Results were then plugged into Eq. (1) to determine GCX coverage. To determine GCX thickness, tissue sample was divided into 4 subcategories: top, bottom, left, and right. Using a random number generator MATLAB code, 3 grid boxes from each subcategory was selected. GCX thickness was measured in each of the 3 selected grid boxes, to obtain a total of 12 GCX thickness measurements per vessel ring. Since 3 vessel rings were examined, a total of 36 measurements were collected per animal per carotid artery to determine GCX thickness. (B) This is a zoomed in image of an examined vessel wall section, which is highlighted in the yellow box of Fig. 1a. A transverse line (yellow) was drawn in the middle of the grid box to assess GCX thickness. (JPG 277 kb)

## Figures and Tables

**Table 1:** Summary of the three cohorts presented in this experiment. All mice were sacrificed after one week of experimental conditions

Group	Mouse-Type	Diet	LCA Ligation
I	Male ApoE-KO	Chow	No (Uniform Flow)
II	Male ApoE-KO	High Fat Diet	No (Uniform Flow)
III	Male ApoE-KO	Chow	Yes (Disturbed Flow)

**Table 2:** Resistance values for each outlet

Resistance Values	LCA, R_LCA_ (dynes. s/cm^5^)	RCA, R_RCA_ (dynes. s/cm^5^)	Descending aorta (DESAO), R_DESAO_ (dynes. s/cm^5^)
Pre-ligation: day 0	4.6597 × 10^4^	4.6597 × 10^4^	6.7228 × 10^3^
Post-ligation: day 1	1.1046 × 10^6^	4.6597 × 10^4^	6.7228 × 10^3^

**Table 3:** Normalized wall shear stresses before and after ligation in the middle portions of the carotid arteries

Normalized wall shear stress	RCA	LCA
Pre-ligation: day 0	0.8856	1.1144
Post-ligation: day 1	1.4539	0.0642

## Abbreviations

ApoE-KO: Apolipoprotein-E-knockout
CD68: Cluster of differentiation 68
CTNI: Center of Translational Neuroimaging
DAPI: 4′,6-diamidino-2-phenylindole
FA: Flip angle
FOV: Field of view
GCX: Glycocalyx
HFD: High fat diet
HRP: Horseradish peroxidase
ICAM-1: Intercellular cell adhesion molecule 1
LCA: Left carotid artery
LDL-R: Low density lipoprotein receptor
MRI: Magnetic resonance imaging
NU-IACUC: Northeastern University Institutional Animal Care and Use Committee
PECAM: Platelet endothelial cell adhesion molecule
RCA: Right carotid artery
TE: Echo time
TR: Repetition time
UTE: Ultra-short echo time
VCAM-1: Vascular cell adhesion molecule 1
WGA: Wheat germ agglutinin
$\bar{\mathrm{WSS}}$: Mean wall shear stress
