# Supplementary figures and images for: The comparative effects of high fat diet or disturbed blood flow on glycocalyx integrity and vascular inflammation
Source: Transl Med Commun. Author manuscript; Available in PMC 2019 Apr 3. (PMC6447085; doi:10.1186/s41231-018-0029-9)

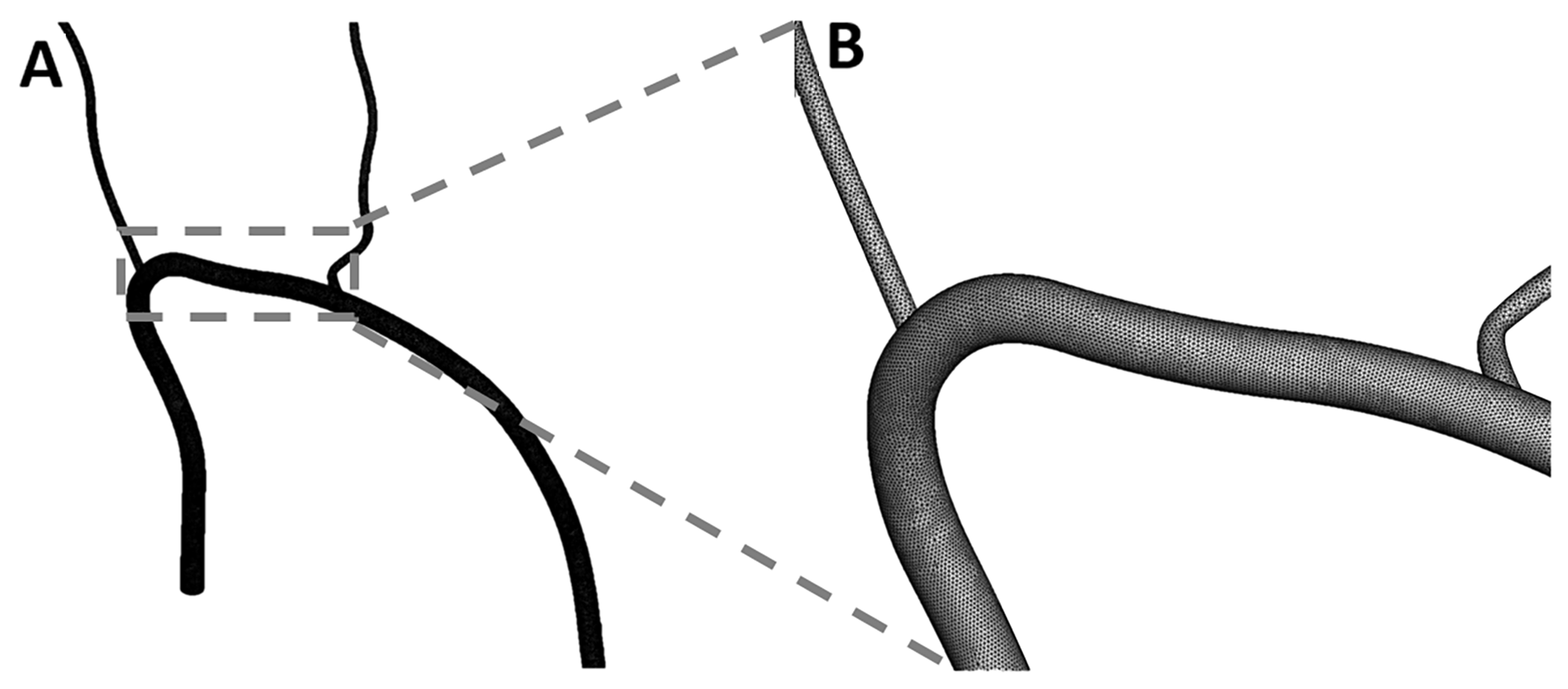

Supplement: Figure S1 — Additional file 1: Figure S1. (A) Perspective view of the discretized, image-based model of the subject-specific vasculature from 1-day LCA post-ligation surgery. (B) Zoomed in view of the computational mesh created. (JPG 94 kb) [file NIHMS998792-supplement-Figure_S1.tif]

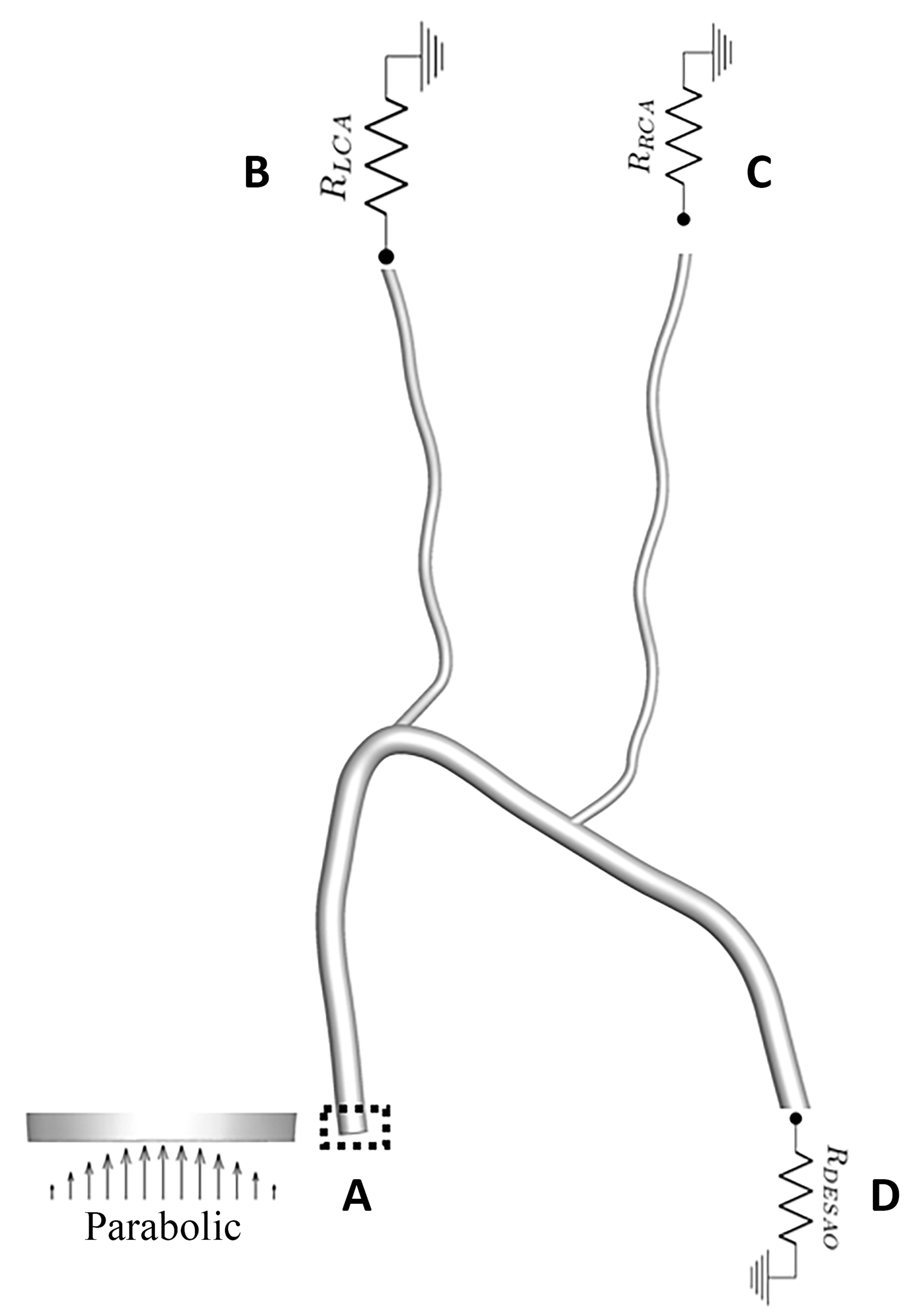

Supplement: Figure S2 — Additional file 2: Figure S2. A schematic of the model from a preligation mouse with corresponding boundary conditions. (A) Parabolic inlet velocity profile. (B), (C), and (D) are resistance outlet conditions at RCA, LCA, and descending aorta respectively. (JPG 165 kb) [file NIHMS998792-supplement-Figure_S2.tif]

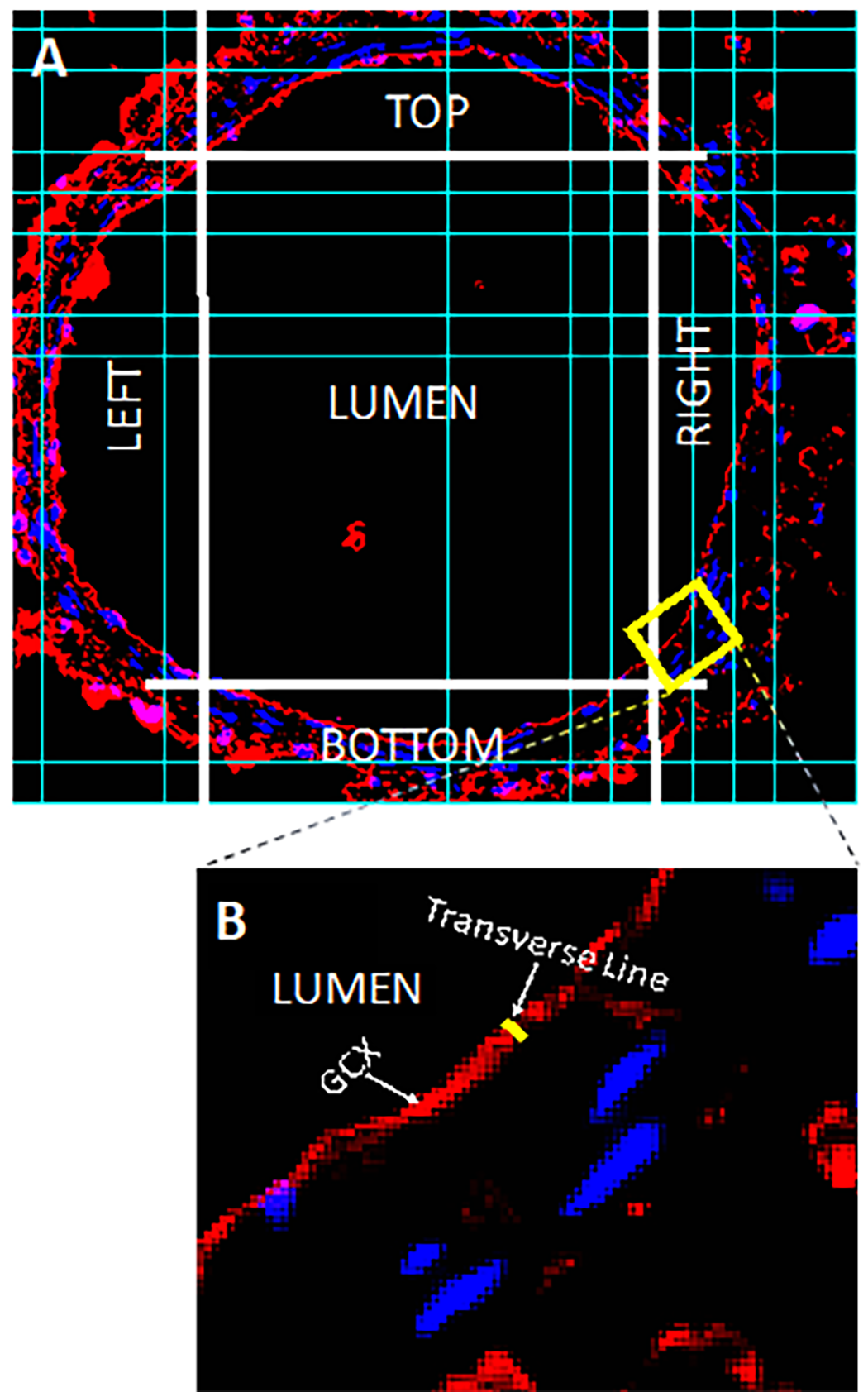

Supplement: Figure S3 — Additional file 3: Figure S3. 20× magnification images of the carotid arteries were used to determine GCX coverage and thickness. The red indicates GCX and the blue represent cell nuclei (DAPI). (A) The initial step was to count the total number of grid boxes encompassing the inner diameter (lumen side) of the arterial wall. Next, the number of grid boxes that contained an incomplete or absent GCX layer was tallied. Results were then plugged into Eq. (1) to determine GCX coverage. To determine GCX thickness, tissue sample was divided into 4 subcategories: top, bottom, left, and right. Using a random number generator MATLAB code, 3 grid boxes from each subcategory was selected. GCX thickness was measured in each of the 3 selected grid boxes, to obtain a total of 12 GCX thickness measurements per vessel ring. Since 3 vessel rings were examined, a total of 36 measurements were collected per animal per carotid artery to determine GCX thickness. (B) This is a zoomed in image of an examined vessel wall section, which is highlighted in the yellow box of Fig. 1a. A transverse line (yellow) was drawn in the middle of the grid box to assess GCX thickness. (JPG 277 kb) [file NIHMS998792-supplement-Figure_S3.tif]
